# Peripheral Cannabinoid-1 Receptor Blockade Ameliorates Cystitis Severity

**DOI:** 10.1089/can.2022.0077

**Published:** 2023-08-09

**Authors:** Liad Hinden, Rami Ludyansky, Sary Leidershnaider, Yoav Harris, Alina Nemirovski, Ofer N. Gofrit, Joseph Tam, Guy Hidas

**Affiliations:** ^1^ Obesity and Metabolism Laboratory, Department of Drug Research, School of Pharmacy, Faculty of Medicine, The Hebrew University of Jerusalem, Jerusalem, Israel.; ^2^ Department of Urology Hadassah Medical Center, Faculty of Medicine, Hebrew University of Jerusalem, Jerusalem, Israel.; ^3^ In partial fulfillment of MD requirements, Hadassah Hebrew University Medical Center, Jerusalem, Israel.

**Keywords:** cannabinoid-1 receptor, cyclophosphamide, cystitis, endocannabinoid system, lower urinary tract symptoms

## Abstract

**Background:**

The endocannabinoid system (ECS) plays a key physiological role in bladder function and it has been suggested as a potential target for relieving lower urinary tract symptoms (LUTSs). Whereas most studies indicate that activating the ECS has some beneficial effects on the bladder, some studies imply the opposite. In this study, we investigated the therapeutic potential of peripheral cannabinoid-1 receptor (CB_1_R) blockade in a mouse model for LUTSs.

**Materials and Methods:**

To this end, we used the cyclophosphamide (CYP; 300 mg/kg, intraperitoneal)-induced cystitis model of bladder dysfunction, in which 12-week-old, female C57BL/6 mice were treated with the peripherally restricted CB_1_R antagonist, JD5037 (3 mg/kg), or vehicle for three consecutive days. Bladder dysfunction was assessed using the noninvasive voiding spot assay (VSA) as well as the bladder-to-body weight (BW) ratio and gene and protein expression levels; ECS tone was assessed at the end of the study.

**Results:**

Peripheral CB_1_R blockade significantly ameliorated the severity of CYP-induced cystitis, manifested by reduced urination events measured in the VSA and an increased bladder-to-BW ratio. Moreover, JD5037 normalized CYP-mediated bladder ECS tone imbalance by affecting both the expression of CB_1_R and the endocannabinoid levels. These effects were associated with the ability of JD5037 to reduce CYP-induced inflammatory response, manifested by a reduction in levels of the proinflammatory cytokine, tumor necrosis factor alpha (TNFα), in the bladder and serum.

**Conclusions:**

Collectively, our results highlight the therapeutic relevance of peripheral CB_1_R blockade in ameliorating CYP-induced cystitis; they may further support the preclinical development and clinical use of peripherally restricted CB_1_R antagonism for treatment of LUTSs.

## Introduction

Lower urinary tract symptoms (LUTSs) include increased frequency, urgency, nocturia, incontinence, recurrent urinary tract infections, and even renal dysfunction.^1^ LUTSs affect ∼16% of the population aged over 40 years and their prevalence increases with age.^2^ LUTSs have a major impact on patients' quality of life due to the tremendous suffering and withdrawal of these individuals from social and professional activities.

The etiology of LUTSs is varied and includes neurogenic conditions (e.g., spinal cord lesions, multiple sclerosis, and diabetic neuropathy), and more commonly, patients present without any defined neurological disorder.^1^ In addition, hemorrhagic cystitis, manifested by dysuria and hematuria as a complication of cyclophosphamide (CYP) therapy, is also largely involved in development of LUTSs in oncological patients.^3^

CYP, a chemotherapeutic drug used to treat different types of cancers, is metabolized to acrolein by the liver and accumulates in the bladder, resulting in hemorrhagic cystitis and LUTSs, especially in immunocompromised patients.^4^ Unfortunately, there is no proven treatment to manage this type of condition, which can severely degrade the patient's quality of life and may possibly lead to renal failure and even death.

The endocannabinoid system (ECS) consists of the two main (most studied) endocannabinoids (eCBs): *N*-arachidonoylethanolamine (anandamide [AEA]) and 2-arachidonoylglycerol (2-AG), and their synthesizing and degrading enzymes, as well as their G-protein-coupled cannabinoid receptors, CB_1_R and CB_2_R. Both cannabinoid receptors have been recently discovered in human and animal bladders, urothelial cells, detrusor muscle, and nerve fibers innervating the bladder.^5–8^

Most studies show the beneficial effects of ECS activation in ameliorating LUTSs (reviewed in Ref.^9^). Specifically, it was reported that intravenous (IV) injection of the AEA transport inhibitor, VDM-11, into anesthetized rats, which increases AEA concentrations, increased the micturition interval and threshold pressure. This effect was blocked by a CB_1_R antagonist (AM251), but not by a CB_2_R antagonist (AM630).^10^ Others have shown that IV injection of a fatty acid amide hydrolase (FAAH) inhibitor into conscious rats also elevates AEA levels and enhances intercontraction intervals, micturition volume, bladder capacity, and threshold pressure.^11^

On the other hand, elevated bladder AEA levels can also activate other off-target receptors such as the transient receptor potential cation channel subfamily V member 1 (TRPV1), which may evoke hyperreflexia and hyperalgesia and increase the micturition volume.^7,12–14^

In recent years, there has been a growing interest in blocking CB_1_R in peripheral organs for treatment of many pathologies mainly associated with metabolic syndrome (reviewed in Refs.^15,16^). Moreover, CB_1_R antagonism was suggested as a potential therapeutic intervention for acute cisplatin-induced renal dysfunction,^17^ a disease associated with enhanced inflammation, oxidative/nitrosative stress, and cell death.

In this study, we show (for the first time) that peripheral CB_1_R antagonism ameliorates CYP-induced cystitis by reducing micturition events, restoring the bladder-to-body weight (BW) ratio, normalizing bladder ECS tone, and reducing inflammation. These results indicate the therapeutic potential of peripherally restricted CB_1_R antagonism against LUTSs.

## Materials and Methods

### Animals

The Institutional Animal Care and Use Committee of The Hebrew University (AAALAC accreditation no. 1285; approval no. MD-19-15994-3) approved the experimental protocol used. Animal studies are reported in compliance with the Animal Research: Reporting of In Vivo Experiments (ARRIVE) guidelines.^18^

### CYP-induced cystitis animal model

To establish the CYP-induced cystitis mouse model, 12-week-old, female C57BL/6 mice were divided into three groups: (1) a control group, treated with vehicle (Veh; 1% Tween 80, and 4% dimethyl sulfoxide [DMSO] in saline, intraperitoneal [IP]) for three consecutive days; (2) CYP + Veh group, treated with Veh on the 1st day, CYP (300 mg/kg, IP) + Veh on the 2nd day, and Veh on the 3rd day; and (3) CYP + JD5037 group, treated with JD5037 (3 mg/kg, IP) on the 1st day, CYP + JD5037 on the 2nd day, and JD5037 on the 3rd day (see the scheme in Fig. 3A).

On the 4th day, mice were euthanized by cervical dislocation under anesthesia, the bladder was removed and weighed, and it was either snap-frozen or fixed in 4% buffered formalin. Trunk blood was collected, and serum was separated and stored at −80°C until processed for biochemical evaluation. The number of samples in each experiment was determined according to tissue availability, which was limited due to the small size of a mouse bladder.

### Voiding spot assay: noninvasive assessment of bladder dysfunction

On the 3rd day, bladder dysfunction was assessed using a noninvasive voiding spot assay (VSA); each mouse was placed in a single cage with an absorbent filter paper and was allowed to move freely for 4 h; during this time, the micturition events were captured and retained as void spots on the paper.

Normal mice tend to urinate only a few times in the cage corner, whereas CYP-treated mice urinate numerous times all over the filter paper. Urine spots were illuminated with ultraviolet (UV) light using the 2UV Transilluminator (UVP, USA), and the number and area of urine spots were analyzed using ImageJ software (NIH, Bethesda, MD).

### Materials

CYP (ENDOXAN) was purchased from Baxter Oncology (Germany). JD5037 was purchased from MedChemExpress (China).

### Serum and bladder tumor necrosis factor alpha analyses

Serum and bladder levels of tumor necrosis factor alpha (TNFα) were measured by an ELISA kit (MHSTA50; R&D Systems) according to the manufacturer's protocol.

### Histopathological analyses

Paraffin-embedded bladder sections (3 μm) from each group were stained with hematoxylin and eosin. Panoramic bladder images were captured with a Zeiss AxioCam ICc5 color camera mounted on a Zeiss Axio Scope A1 light microscope at ×5 and ×40 magnifications.

### Immunohistochemistry

Bladder tissues from Veh- and CYP-injected mice (five animals per group) were fixed in 4% buffered formalin for 48 h and then embedded in paraffin. Sections were deparaffinized and hydrated. Heat-mediated antigen retrieval was performed with 10 mM citrate buffer, pH 6.0 (Thermo Scientific, IL, USA). Endogenous peroxide was inhibited by incubating with a freshly prepared 3% hydrogen peroxide (H_2_O_2_) solution in methanol (MeOH).

Unspecific antigens were blocked by incubating sections for 1 h with 2.5% horse serum (VE-S-2000; Vector Laboratories). Next, 3-μm bladder sections were stained for rabbit-anti-CB_1_R (ACR-001; Alomone), followed by a goat anti-rabbit horseradish peroxidase (HRP) conjugate (ab97085; Abcam). Color was developed after incubation with 3,3′-diaminobenzidine (DAB) substrate (SK-4105, ImmPACT DAB Peroxidase [HRP] Substrate; Vector Laboratories), followed by hematoxylin counterstaining and mounting (VectaMount H-5000; Vector Laboratories).

Stained sections were photographed as described above. Positive areas were quantified using ImageJ software with a minimum of four random images of the detrusor muscle or urothelium per mouse.

### Western blotting

Bladder homogenates were prepared in RIPA buffer (25 mM Tris-HCl, pH 7.6; 150 mM NaCl; 1% NP-40; 1% sodium deoxycholate; and 0.1% sodium dodecyl sulfate [SDS]) using the Bullet Blender^®^ and zirconium oxide beads (Next Advance, Inc., NY, USA). Protein concentrations were measured with a Pierce™ BCA Protein Assay Kit (Thermo Scientific). Samples were resolved by SDS-polyacrylamide gel electrophoresis (PAGE) (4–15% acrylamide, 150V) and transferred to polyvinylidene difluoride (PVDF) membranes using the Trans-Blot^®^ Turbo™ Transfer System (Bio-Rad, CA).

Membranes were then incubated for 1 h in 5% milk (in 1× Tris-buffered saline with 0.1% Tween^®^ 20 detergent [TBS-T]) to block unspecific binding and then incubated overnight with rabbit anti-CB_1_R (#301214; Immunogen) and TRPV1 (#ACC-029; Alomone) antibodies at 4°C. Anti-rabbit HRP-conjugated secondary antibody (#97085; Abcam) was used for 1 h at room temperature, followed by chemiluminescence detection using Clarity™ Western ECL Blotting Substrate (Bio-Rad).

Densitometry was quantified using Bio-Rad CFX Manager software. Quantification was normalized to the anti-β-actin antibody (#ab49900; Abcam).

### Real-time PCR

Total bladder messenger RNA (mRNA) was extracted using Bio-Tri RNA lysis buffer (Bio-Lab, Israel), followed by DNase I treatment (Thermo Scientific), and reverse transcribed using the iScript cDNA kit (Bio-Rad). Real-time PCR was performed using the iTaq Universal SYBR Green Supermix (Bio-Rad) and the CFX connect system (Bio-Rad).

The primers used to detect mouse genes are listed in Table 1. Mouse genes were normalized to *Ubc*.

**Table 1.** Mouse Primers Used for Real-Time PCR Analysis

Gene	Forward primer (5′-3′)	Reverse primer (5′-3′)
*Cnr1*	AAGTCGATCTTAGACGGCCTT	TCCTAATTTGGATGCCATGTCTC
*Cnr2*	CTGCAGCTCTTGGGACCTAC	TGTCCCAGAAGACTGGGTGT
*Col1*	TTCTCCTGGCAAAGACGGACTCAA	GGAAGCTGAAGTCATAACCGCCA
*Col3*	ACAGCAAATTCACTTACACAGTTC	CTCATTGCCTTGCGTGTTT
*Daglb*	AGCGACGACTTGGTGTTCC	GCTGAGCAAGACTCCACCG
*Faah*	GTATCGCCAGTCCGTCATTG	GCCTATACCCTTTTTCATGCCC
*Fn1*	ATGTGGACCCCTCCTGATAGT	GCCCAGTGATTTCAGCAAAGG
*Cxcl10*	GGATGGCTGTCCTAGCTCTG	TGAGCTAGGGAGGACAAGGA
*Il18*	GACTCTTGCGTCAACTTCAAGG	CAGGCTGTCTTTTGTCAACGA
*Ccl2*	GCATTAGCTTCAGATTTA	TTAAAAACCTGGATCGGAACCAA
*Mgll*	ACCATGCTGTGATGCTCTCTG	CAAACGCCTCGGGGATAACC
*Napepld*	ACGTCCTCCTCTAGTCTGTAATC	AGCGCCAAGCTATCAGTATCC
*Tgfb*	GCGGACTACTATGCTAAAGAGG	GTAGAGTTCCACATGTTGCTCC
*Ubc*	CAGCCGTATATCTTCCCAGAC	CTCAGAGGGATGCCAGTAATC
*Tnfa*	QT00104006 QuantiTect Primer Assays (Qiagen, Germany)	

*Ccl2* (Mcp1), mast cell proteinase; *Cnr*, cannabinoid receptor; *Col*, collagen; *Cxcl10* (Ip10), C-X-C motif chemokine ligand 10; *Dagl*, diacylglycerol lipase; *Faah*, fatty acid amide hydrolase; *Fn*, fibronectin; *Il*, interleukin; *Mgll*, monoacylglycerol lipase; *Napepld*, N-acyl phosphatidylethanolamine phospholipase D; *Tgf*, transforming growth factor; *Tnf*, tumor necrosis factor; *Ubc*, ubiquitin C.

### Sample preparation and endocannabinoid measurements by liquid chromatography with tandem mass spectrometry

eCBs were extracted, purified, and quantified from bladder lysates. In brief, bladders were homogenized in ice-cold Tris buffer using the Bullet Blender and zirconium oxide beads (Next Advance, Inc.); protein concentration was determined by the bicinchoninic acid (BCA) assay. Samples were then supplemented with an ice-cold extraction buffer [1:1 methanol/Tris buffer + an internal standard (IS)] and chloroform/methanol (2:1), vortexed, and centrifuged.

The lower organic phase was transferred into borosilicate tubes; this step was repeated three times by adding ice-cold chloroform to the samples and transferring the lower organic phase into the same borosilicate tubes. The samples were dried and kept overnight at −80°C, then reconstituted with ice-cold chloroform and acetone, kept at −20°C for 30 min, and then centrifuged to precipitate proteins.

Next, the supernatant was dried and reconstituted in an ice-cold liquid chromatography with tandem mass spectrometry grade methanol and analyzed on an AB Sciex (Framingham, MA) QTRAP^®^ 6500 + mass spectrometer coupled with a Shimadzu (Kyoto, Japan) ultra high-performance liquid chromatography (UHPLC) System. Liquid chromatographic separation was achieved using 5-μL injections of samples into a Kinetex 2.6-μm C18 (100×2.1 mm) column from Phenomenex (Torrance, CA). The autosampler was set at 4°C, and the column was maintained at 40°C during the entire analysis.

The gradient elution mobile phases consisted of 0.1% formic acid in water (phase A) and 0.1% formic acid in acetonitrile (phase B). eCBs were detected in a positive ion mode using electron spray ionization and the multiple reaction monitoring mode of acquisition, using d_4_-AEA as IS. The collision energy, declustering potential, and collision cell exit potential for the monitored transitions are presented in Table 2.

**Table 2.** Multiple Reaction Monitoring Transitions for Endocannabinoid Measurements in Electron Spray Ionization (ESI)+ and ESI−

Analyte	Molecular ion [M+H]^+^[M–H]^−^ for AA (m/z)	Fragment (m/z)	DP (volts)	CE (volts)	CXP (volts)
2-AG	379.2	287.1 (quantifier)	70	19	14
		91 (qualifier)	70	67	10
AEA	348.2	287.1 (quantifier)	26	13	16
		62 (qualifier)	26	13	8
d_4_-AEA	352.3	287.1 (quantifier)	66	15	20
		66 (qualifier)	66	21	8

2-AG, 2-arachidonoylglycerol; AEA, anandamide; CE, collision energy; CXP, collision cell exit potential; DP, declustering potential.

The levels of AEA and 2-AG in the samples were measured against standard curves and normalized to the bladder lysate protein concentration.

### Statistics

Values are expressed as the mean±standard error of mean (SEM). Unpaired two-tailed Student's *t*-test was used to determine differences between the two groups. Results of multiple groups were compared by one-way analysis of variance, followed by one-sided Tukey's test, using GraphPad Prism, v6 for Windows (San Diego, CA). Significance was set at *p*<0.05.

## Results

### CYP-induced cystitis exacerbates micturition events and bladder inflammation

**FIG. 1.**
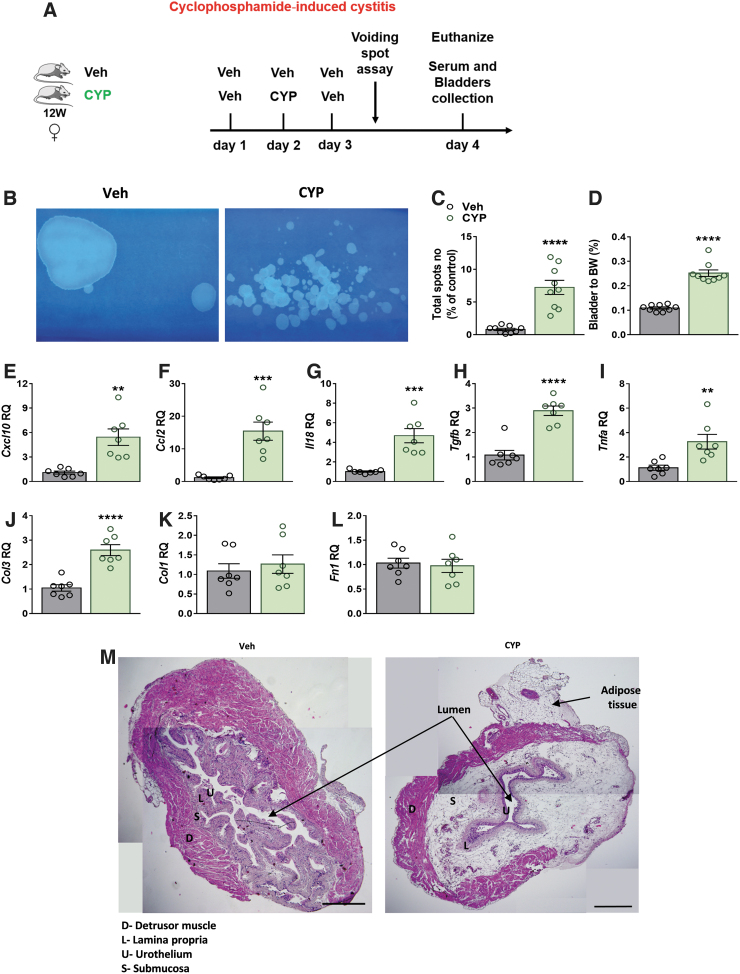
CYP-induced cystitis exacerbates micturition events and bladder inflammation. CYP-induced cystitis—experimental design **(**see the Materials and Methods section; **A)**. CYP-induced cystitis elevated micturition events **(B, C)**, bladder-to-BW ratio **(D)**, and bladder mRNA expression levels of inflammatory markers **(E–I)**. The mRNA levels of fibrotic markers showing elevation in expression of *Col3* **(J)**, but not *Col1* and *Fn1* **(K, L)**, following injection of CYP. Representative panoramic H&E staining of bladders from each group, original magnification×5, scale bar—500 μm **(M)**. Data represent the mean±SEM. For **(C, D)**, *n*=9 mice per group, and for **(E–L)**, *n*=7 mice per group. **p<0.01, ***p<0.001, and ****p<0.0001. BW, body weight; CYP, cyclophosphamide; H&E, hematoxylin and eosin; mRNA, messenger RNA; SEM, standard error of mean; Veh, vehicle.

To evaluate the severity of CYP-induced cystitis in our mouse model (Fig. 1A), noninvasive VSA was conducted. Significant increases in urine spots (Fig. 1B, C) and the bladder-to-BW ratio (Fig. 1D) were found in the CYP-injected mice. The cystitis was associated with enhanced bladder mRNA expression levels of the inflammatory cytokines, *Cxcl10*, *Ccl2*, *Il18*, *Tgfb*, and *Tnfa* (Fig. 1E–I).

In contrast, expression levels of only the fibrotic marker *Col3* (Fig. 1J), and not *Col1* and *Fn1* (Fig. 1K, L), were enhanced following CYP injection, as expected from an acute model. Nevertheless, histological examination of bladders revealed a typical appearance of cystitis with excessive submucosal edema and hemorrhage (Fig. 1M).

### CYP-induced cystitis upregulates CB_1_R and TRPV1 expression

**FIG. 2.**
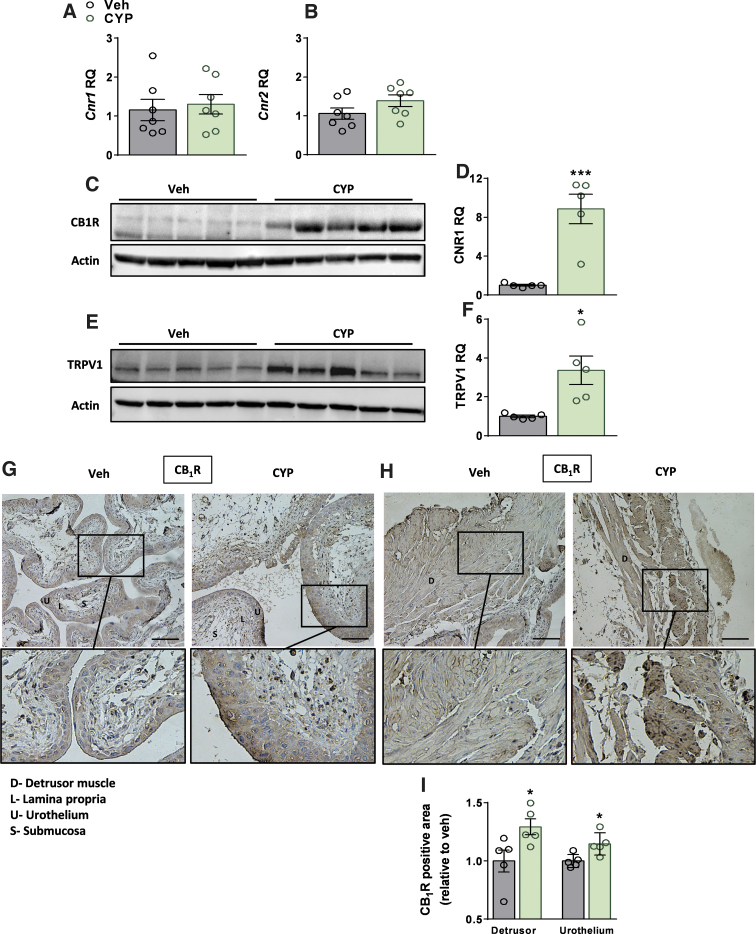
CYP-induced cystitis upregulates CB_1_R and TRPV1 expression. No significant changes in the bladder mRNA expression levels of *Cnr1* or *Cnr2* in CYP-injected mice **(A, B)**. Significant elevation in the bladder protein expression levels of CB_1_R **(C, D)** and TRPV1 **(E, F)**. Representative immunohistochemical staining of CB_1_R in the bladder's urothelial cells **(G)** and detrusor muscle **(H)** and quantification of CB_1_R-positive areas in these cells **(I)** for each group, magnification×40, scale bar—50 μm. Data represent the mean±SEM. For **(A, B)**, *n*=7 mice per group, and for **(D, F, I)**, *n*=5 mice per group. *p<0.05 and ***p<0.001. CB_1_R, cannabinoid-1 receptor; TRPV1, transient receptor potential cation channel subfamily V member 1.

CYP-induced cystitis did not significantly change the mRNA expression levels of *Cnr1* or *Cnr2* (Fig. 2A, B); however, the protein expression levels of CB_1_R were markedly elevated (Fig. 2C, D), indicating an elevated ECS tone. CB_2_R protein levels were not examined due to the lack of a reliable antibody for this receptor. Since the ECS can also activate other off-target receptors, such as the TRPV1, we examined its expression and detected significant upregulation in its protein expression levels (Fig. 2E, F) in the CYP-treated mice.

Elevated CB_1_R protein expression levels were further validated using immunohistochemistry, revealing significant elevated expression levels in urothelial cells, particularly in the umbrella cells, and the lamina propria, as well as in detrusor muscle cells (Fig. 2G–I).

### Peripherally restricted CB_1_R antagonism attenuates CYP-induced cystitis and inflammation

**FIG. 3.**
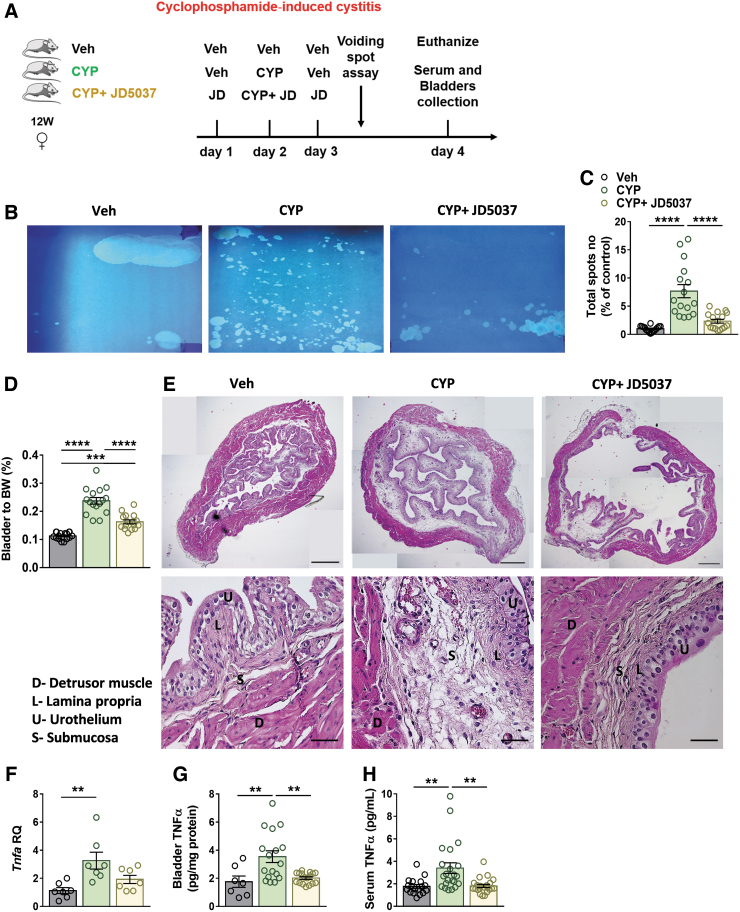
Peripherally restricted CB_1_R antagonism attenuates CYP-induced cystitis severity and inflammation. A CYP-induced cystitis model with CB_1_R antagonism—experimental design **(**see the Materials and Methods section; **A)**. Treatment with the peripherally restricted CB_1_R antagonist, JD5037 (3 mg/kg, IP), significantly reduced micturition events **(B, C)**, the bladder-to-BW ratio **(D)**, and submucosal edema and hemorrhage, as shown in the representative panoramic H&E staining of bladders from each group, (original magnification×5, scale bar—500 μm; and×40 scale bar—50 μm; **E)**, as well as normalized the elevated bladder mRNA **(F)** and protein **(G)** levels of TNFα and its circulating levels **(H)**. Data represent the mean±SEM. For **(C)**, *n*=16 mice per group; for **(D)**, *n*=27 mice per group; for **(F)**, *n*=7 mice per group; for **(G)**, *n*=7 mice for the Veh group and 17 mice for the CYP-treated groups; and for **(H)**, *n*=18 mice for the Veh group and 22 mice for the CYP-treated groups. **p<0.01, ***p<0.001, and ****p<0.0001. CYP, cyclophosphamide; IP, intraperitoneal; JD, JD5037; TNFα, tumor necrosis factor alpha; Veh, vehicle.

Treatment with the peripherally restricted CB_1_R antagonist, JD5037, a day before, in combination with, and a day after CYP injection (Fig. 3A) significantly reduced the elevated urine spots (Fig. 3B, C) and the bladder-to-BW ratio (Fig. 3D) in CYP-injected mice. Histologically, JD5037 reduced the area affected by edema and hemorrhage (Fig. 3E).

Moreover, CB_1_R antagonism resulted in normalization of elevated mRNA (Fig. 3F) and protein (Fig. 3G) levels of the inflammatory cytokine, TNFα, as well as its circulating levels in the serum (Fig. 3H). Taken together, these results indicate that peripheral CB_1_R antagonism attenuates cystitis severity.

### Peripherally restricted CB_1_R antagonism normalizes bladder ECS tone

**FIG. 4.**
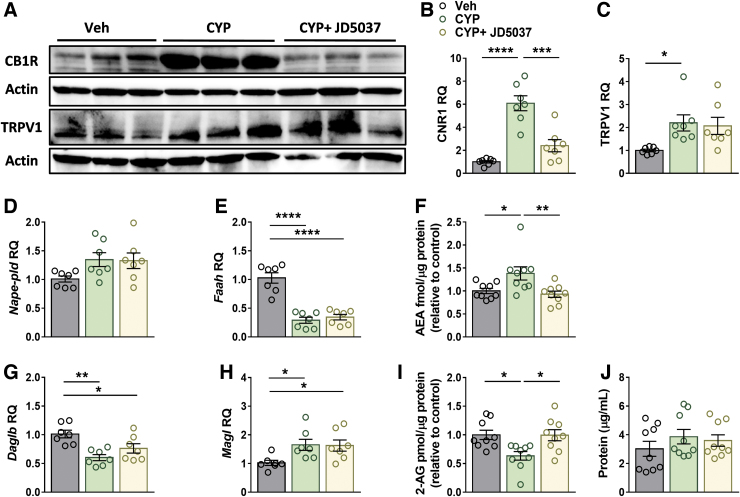
Peripherally restricted CB_1_R antagonism restores the ECS tone. JD5037 treatment restored bladder protein expression levels of CB_1_R **(A, B)** in CYP-injected mice, but not the elevated protein levels of TRPV1 **(A, C)**, as shown in the representative immunoblots. CYP did not induce any significant changes in the mRNA expression levels of *Nape-pld* **(D)**; however, it did induce a significant reduction in *Faah* **(E)**. Accordingly, the actual amount of AEA increased in the CYP-injected mice and was normalized by JD5037 **(F)**. CYP induced a significant reduction in the bladder mRNA levels of *Daglb* **(G)** and significant upregulation of *Magl* (**H**). Accordingly, the actual amount of 2-AG was significantly reduced in the CYP-injected mice and normalized by JD5037 **(I)**. Bladder protein concentrations **(J)**. Data represent the mean±SEM. For **(B–E** and **G, H)**, *n*=7 mice per group, and for **(F, I, J)**, *n*=9 mice per group. *p<0.05, **p<0.01, ***p<0.001, and ****p<0.0001. 2-AG, 2-arachidonoylglycerol; AEA, anandamide; *Daglb*, diacylglycerol lipase b; ECS, endocannabinoid system; *Faah*, fatty acid amide hydrolase; *Magl*, monoacylglycerol lipase; *Nape-pld*, N-acyl phosphatidylethanolamine phospholipase D.

To assess the involvement of the ECS in CYP-induced cystitis and its reversal by JD5037, we assessed changes in expression of the receptors and ligands in this system in our model. Interestingly, the improvements in bladder function by JD5037 were accompanied by downregulation of CB_1_R (Fig. 4A, B), but not the TRPV1 expression levels (Fig. 4A, C).

We next determined the mRNA levels of degrading and synthesizing enzymes as well as the amount of AEA and 2-AG in the bladder. Whereas CYP-injected mice displayed no significant changes in the expression levels of AEA's synthesizing enzyme, *N*-acyl phosphatidylethanolamine phospholipase D (Fig. 4D), a significant reduction in the expression of its degrading enzyme, FAAH, (Fig. 4E) was measured.

These changes were found to be in accordance with the actual amount of AEA found in the bladder (Fig. 4F). Interestingly, JD5037 treatment normalized AEA levels in the treated mice (Fig. 4F), although it did not affect the expression of its related enzymes. In contrast to AEA, decreased synthesis and enhanced catabolism of 2-AG, resulting in reduced bladder levels, were found in mice injected with CYP, as manifested by changes in expression levels of 2-AG's synthesizing enzyme, diacylglycerol lipase beta, and its degrading enzyme, monoacylglycerol lipase (Fig. 4G, H), respectively.

Yet, similar to the effect of JD5037 on AEA levels, the treatment also normalized the amount of 2-AG in the bladder (Fig. 4I) without affecting the expression of its related enzymes. Both AEA and 2-AG levels were normalized to the protein levels in each bladder (Fig. 4J), which were not affected by CYP or JD5037 treatment. These findings indicate that peripheral CB_1_R antagonism can restore bladder ECS tone.

## Discussion

The present study reveals, for the first time, the relevance of CB_1_R antagonism in ameliorating hemorrhagic cystitis. We found that peripheral CB_1_R antagonism reduces the elevated micturition events, bladder edema, and inflammation as well as restores the bladder ECS tone. Our findings are unique since most publications in this field indicate that activation of CB_1_R or CB_2_R is an effective strategy to decrease LUTSs in animal models (reviewed in Ref.^9^).

These studies mostly refer to the modulatory action of CB_1_R on the sensory neurons innervating the bladder as well as to the anti-inflammatory effect of CB_2_R agonism. In contrast, our findings suggest an alternative pathway by which bladder dysfunction can be mitigated through reducing the enhanced activity of the CB_1_R present on urothelial cells^5,8^ and the detrusor muscle.^8^

The ECS, ubiquitously present in humans and animals, acts both centrally and peripherally to maintain cellular and organ homeostasis. Thus, changes in ECS tone, evidenced by modulation in expression of the cannabinoid receptors, their functional activity (upregulated or downregulated), and the relative number of eCBs, may render the subject susceptible to different diseases.^19^

The analgesic effects of CB_1_R agonists are well established in many chronic disorders.^20^ Upregulation of CB_1_R during cystitis revealed here could be one of the compensatory mechanisms to ameliorate pain and inflammation in acute hemorrhagic cystitis in mice. Indeed, bladder cystitis is characterized by an imbalanced ECS tone, as manifested here by enhanced bladder expression of CB_1_R and altered AEA and 2-AG levels, as well as changes in their corresponding synthesizing and degrading enzymes.

These findings are in accordance with those reporting elevated AEA levels and bladder inflammation in rats, but not in mouse models for cystitis.^14,21,22^ However, in contrast to others, they showed no changes in 2-AG levels in a CYP-induced mouse model for cystitis.^22^ These discrepancies may be related to differences in the model established, the number of mice used in each experiment, the eCB extraction method, and the normalization method used for calculating eCB levels.

Regarding eCB levels, we used normalization to the bladder's protein content rather than the bladder weight since its increased weight could arise from edema and water absorption, resulting in higher chances for errors in calculating the eCB levels. Nevertheless and surprisingly, we found that peripheral CB_1_R blockade results in a modulatory effect on the ECS, and normalizes its action under these conditions, both regarding the receptor expression levels and the amount of ligands produced in the bladder.

Although CB_1_R blockade normalized AEA and 2-AG levels, the mRNA expression levels of their synthesizing and degrading enzymes were not significantly changed; these contradicting results may be explained by changes in their protein expression levels or their activities. Future experiments would need to assess these assumptions experimentally.

In attempting to determine the specific effects of AEA and 2-AG on bladder function, we suggest that the two ligands may play opposing roles. Whereas some studies imply that AEA increases the micturition interval and threshold pressure^10,11^ and may decrease pain behavior and bladder hypersensitivity,^21,22^ others have shown that elevated bladder AEA levels may activate pronociceptive TRPV1 channels^7,14,23,24^ and induce pain.

Our results support the latter observations since we found that CYP elevates AEA levels and TRPV1 expression. In fact, normalization of AEA levels by JD5037 therefore has great importance in ameliorating pain sensation and further suggests that peripheral CB_1_R blockade may have an indirect analgesic effect in this model; however, this hypothesis should be further tested experimentally with behavioral and pain tests.

Regarding the role of 2-AG in the bladder and its opposite pattern to AEA, reduced inflammation in CB_1_R antagonist-treated mice may be due to the recovery of 2-AG (preferential a CB_2_R ligand) levels, and it may support the anti-inflammatory role of CB_2_R that was well established in cystitis.^25,26^ Since we have not measured the protein expression of CB_2_R (due to lack of a valid antibody), we cannot comment further on its role in our settings.

Peripheral CB_1_R overactivation is strongly related to metabolic inflammation^27^ and its peripheral antagonism has great therapeutic potential in reducing it. Specifically, CB_1_R activation has been associated with increased TNFα secretion in human bladder carcinoma cells,^28^ whereas CB_1_R antagonism was reported to inhibit TNFα production in the inflamed small intestine in rats.^29^

Moreover, CB_1_R genetic deletion reduces TNFα secretion by Kupffer cells,^30^ and pharmacological CB_1_R antagonism or its genetic deletion inhibits TNFα expression in diabetic cardiomyopathy.^31^ Moreover, we have previously shown that peripheral CB_1_R antagonism or its genetic deletion from renal proximal tubule cells normalizes the elevated TNFα levels in both type 1 diabetes and diet-induced obesity mouse models.^32,33^ In agreement with all of these studies, we show here that peripheral CB_1_R antagonism also normalizes bladder TNFα levels as well as systemic inflammation.

Although CYP-induced cystitis is not defined as a chronic metabolic disease, there is ample evidence that acute pathologies are also accompanied by an imbalanced ECS tone and enhanced CB_1_R activity.^17,34^ In fact, this upregulation may be manifested as a compensatory effect by the damaged organ to restore its function. However, this imbalance may promote unwanted effects such as a destructive inflammatory response.

In conclusion, hemorrhagic cystitis is characterized by bladder dysfunction, inflammation, and altered ECS tone. Peripheral CB_1_R blockade has the potential to ameliorate these characteristics and to restore normal bladder function. These findings support the rationale for clinical testing of peripheral CB_1_R blockers that are currently being developed in preclinical settings for the treatment of CYP-induced cystitis in oncologic patients as well as other systemic conditions associated with LUTSs.

## Abbreviations Used

2-AG: 2-arachidonoylglycerol
AEA: anandamide
BW: body weight
CB_1_R: cannabinoid-1 receptor
CE: collision energy
CXP: collision cell exit potential
CYP: cyclophosphamide
DAB: 3,3′-diaminobenzidine
DAGLB: diacylglycerol lipase b
DP: declustering potential
eCBs: endocannabinoids
ECS: endocannabinoid system
FAAH: fatty acid amide hydrolase
HRP: horseradish peroxidase
IS: internal standard
LUTSs: lower urinary tract symptoms
MAGL: monoacylglycerol lipase
mRNA: messenger RNA
NAPE-PLD: N-acyl phosphatidylethanolamine phospholipase D
TNFα: tumor necrosis factor alpha
TRPV1: transient receptor potential cation channel subfamily V member 1
VSA: voiding spot assay
